# Phenolic Composition Influences the Health-Promoting Potential of Bee-Pollen

**DOI:** 10.3390/biom9120783

**Published:** 2019-11-26

**Authors:** Mirjana Mosić, Jelena Trifković, Irena Vovk, Uroš Gašić, Živoslav Tešić, Branko Šikoparija, Dušanka Milojković-Opsenica

**Affiliations:** 1 University of Belgrade—Faculty of Chemistry P.O. Box 51, 11158 Belgrade, Serbia; mosicm@chem.bg.ac.rs (M.M.); jvelicko@chem.bg.ac.rs (J.T.); ztesic@chem.bg.ac.rs (Ž.T.); 2 Department of Food Chemistry, National Institute of Chemistry, Hajdrihova 19, SI-1000 Ljubljana, Slovenia; irena.vovk@ki.si; 3 Institute for Biological Research “Siniša Stanković”—National Institute of Republic of Serbia, University of Belgrade, Bulevar despota Stefana 142, 11060 Belgrade, Serbia; 4 BioSense Institute—Research Institute for Information Technologies in Biosystems, University of Novi Sad, 21000 Novi Sad, Serbia; sikoparijabranko@biosense.rs

**Keywords:** bee-pollen, HPTLC, UHPLC-MS/MS, phenolic profile, antioxidant activity

## Abstract

Information on compositional, nutritional and functional properties of bee-pollen, as a health-promoting food, is essential for defining its quality. Concerning the nutritional importance of phenolic compounds, the aim of this study was to determine the phenolic profile and antioxidant activity of twenty-four bee-pollen samples collected from different regions of Serbia. High-performance thin-layer chromatographic (HPTLC) fingerprinting was used for profiling of bee-pollen samples according to the botanical type. HPTLC hyphenated with image analysis and a pattern recognition technique confirmed the grouping of samples caused by the specific phenolic composition of pollens of different botanical origin. Flavonoid glycosides in bee-pollen samples were identified by applying ultra-high-performance liquid chromatography (UHPLC) coupled with linear ion trap-Orbitrap mass spectrometry (LTQ Orbitrap MS). Eight out of twenty-seven flavonol glycosides were identified in bee-pollen samples for the first time. All analyzed bee-pollen samples showed a high number of phenolic compounds which may have therapeutic potential.

## 1. Introduction

Bee-pollen is the main source of important nutrients and phytochemicals that honeybees collect for the purpose of feeding their larvae. It is a mixture of floral pollens, nectar and mouth secretions of bees, accumulated as pellets of different color, size and morphology. Due to its beneficial effect in the human diet, bee-pollen is considered as a health-promoting food. Inter alia, these effects are related to the antioxidant properties of the phenolic compounds present in pollen [1]. They have a role in the protection of vital cell components from oxidative damage by neutralizing free radicals and the prevention of various diseases such as cancer and cardiovascular and neurodegenerative diseases [2]. Phenolic compounds are also recognized as potentially useful taxonomic markers [3]. For this reason, the characterization of phenolic compounds in bee-pollen has been of increasing interest in recent years.

Recent papers have reported that bee-pollen usually contains flavonoid glycosides, particularly derivatives of kaempferol, quercetin, and isorhamnetin, as well as hydroxycinnamic acid derivatives [4]. However, due to the high diversity of phenolic substances, quantitative determination of individual flavonoid glycosides is difficult. For example, liquid chromatography (LC) coupled with mass spectrometry (MS) has been applied in recent research for the identification and quantification of flavonol glycosides in bee-pollen [4,5,6,7,8], red spice paprika from Serbia [9], as well as in *Lathyrus cicera* L. seeds [10]. Using advanced LC-MS techniques, the structures of compounds, for which standards are not commercially available, could be determined by the study of their characteristic fragmentation patterns thanks to the great number of rules for structural characterization that were summarized in the past [11,12,13]. Moreover, tandem MS experiments provide more fragmentations, giving additional structural information for the identification of different classes of phenolic compounds [14].

The antioxidant capacity of a number of bee-pollen samples from different regions of the world has been determined. There are lots of methods appropriate for assessing antioxidant activity, e.g., 2,2-diphenyl-1-picryhydrazyl· (DPPH·) assay, enzymatic or non-enzymatic measurements of lipid peroxidation inhibition, the ferric reducing antioxidant power (FRAP) assay, or the Trolox equivalent antioxidant capacity (TEAC) assay [4,15,16,17].

Due to the high nutritional value and pronounced health-promoting properties, and the potential use as a supplement to the human diet, bee-pollen represents a valuable natural product. However, in the absence of clinical studies that would substantiate the medicinal properties, any new information about the biological activity of bee-pollen is important. Also, a large variability of the chemical composition of bee-pollen complicates the determination of parameters of authenticity, as well as the determination of their biological activity. In our previous publications on the characterization of bee-pollen samples from Serbia, their physicochemical characteristics, techno-functional properties, mineral content and aflatoxin contamination were described [18,19,20]. As a continuation of these studies, the aims of this work were to analyze the phenolic composition of Serbian bee-pollen samples using high-performance thin-layer chromatographic (HPTLC) and ultra-high-performance liquid chromatography (UHPLC) coupled with linear ion trap-Orbitrap mass spectrometry (UHPLC–LTQ Orbitrap MS) techniques. These techniques have already been proven to be reliable for the unambiguous detection of various phenolic compounds in different samples and matrices. In addition, authentication of bee-pollen samples was performed by application of appropriate data evaluation procedures.

## 2. Materials and Methods

### 2.1. Chemicals and Materials

The compound 2-aminoethyl diphenylborinate (NTS), 2,2-diphenyl-1-picrylhydrazyl (DPPH) and flavonoid standards (myricetin, naringenin, chrysin, and galangin) were purchased from Fluka (Steinheim, Germany); acetonitrile and formic acid (both of them MS grade), methanol (HPLC grade), sodium carbonate, hydrochloric acid, toluene, ethyl acetate, and Folin–Ciocalteu reagent from Merck (KGaA, Darmstadt, Germany); polyethyleneglycol 4000 (PEG), standards of phenolic compounds (rutin, hyperoside, astragalin, chlorogenic acid, caffeic acid, gallic acid, and ferulic acid) and Trolox standard from Sigma-Aldrich (Steinheim, Germany). All chemicals which purity is not previously emphasized were of analytical purity grade.

Methanolic standard mixture of phenolic compounds was prepared with following final standard concentrations: galangin (50 ng/µL), chlorogenic acid, myricetin, chrysin and caffeic acid (100 ng/µL), gallic acid, ferulic acid and naringenin (200 ng/µL).

The cartridges for solid-phase extraction (SPE) were Strata C18–E (500 mg/3 mL) obtained from Phenomenex (Torrance, CA, USA). Ultrapure water was obtained from a TKA MicroPure (Thermo Fisher Scientific, Bremen, Germany) water purification system, 0.055 µS/cm. Syringe filters (13 mm, polyfluorotetraethylene (PTFE) membrane 0.45 µm) were purchased from Supelco (Bellefonte, PA, USA).

### 2.2. Bee-Pollen Samples

A total of 24 samples of bee-pollen collected in different locations in Serbia were provided by ‘‘The Federation of the Beekeeping Organizations of Serbia’’ (SPOS) (www.spos.info). The frequency of pollen types in bee-pollen sample was determined and reported in previous study [18]. Two additional bee-pollen samples (marked as **P17** and **P24**) were analyzed in this study following the methodology of pollen analysis described in Kostić et al. [18]. For ease of understanding the results obtained, a list of investigated samples with results of pollen analysis is presented in the Supplementary Materials (Table S1).

### 2.3. Preparation of Bee-Pollen Extracts

Bee-pollen samples (1 g) were suspended in 10 mL of methanol/water (7:3, *v*/*v*) acidified with 0.1 mL of concentrated formic acid (5 g/100 g)) (7:3, *v*/*v*), ultrasonicated for 1 h and centrifuged at 4500 rpm. The supernatant was separated, and re-extraction was performed with the solid residue. The extracts were pooled, and the methanol was evaporated under vacuum at 40 °C until the entire solvent was removed. Then, HCl solution (0.1 g/100 g) was added to the aqueous extract to final volume of 10 mL. In order to remove sugars and polar substances other than phenolic, 2 mL of prepared solution was cleaned up and concentrated by sorption on a SPE cartridge. The cartridge was previously activated according to the procedure described in Waisi et al. [21]. Elution of the phenolic fraction was performed with 1.5 mL acidified methanol (0.1% HCl). Afterwards, this fraction was dissolved (1:10, *v*/*v*) in acetonitrile/water (1:1, *v*/*v*). The obtained solutions were stored at –20 °C. Prior to HPTLC and UHPLC–LTQ Orbitrap MS analysis, bee-pollen extracts were filtered by a 0.45 µm PTFE membrane filter.

### 2.4. Determination of the Total Phenolic Content

The total phenolic content (TPC) was estimated spectrophotometrically according to the Folin–Ciocalteu method [22]. A total of 2.5 mL of Folin–Ciocalteu reagent was added to 0.5 mL of each pollen extract and left at room temperature for 5 min, after which 2 mL of sodium carbonate solution (7.5 g/100 mL) was added. After incubation in the dark for 2 h at 25 °C, absorbance was measured at 765 nm against a blank (the same mixture without the sample) using a Cintra 6 spectrophotometer (GBC Scientific Equipment Ltd., Dandenong, Australia). Gallic acid (20–100 µg/mL) was used as the standard and TPC was expressed as the mg of gallic acid equivalent (GAE) per gram of bee-pollen sample.

### 2.5. High-Performance Thin-Layer Chromatography

Chromatographic separation was performed on silica gel HPTLC plates (20 × 10 cm, Art. 5641, Merck, Darmstadt, Germany). Bee-pollen extracts (2 μL) were applied (as 8 mm bands by an Automatic Thin-layer chromatography (TLC) sampler 4 (ATS4, CAMAG, Muttenz, Switzerland). The composition of mobile phases was as follows: toluene/ethyl acetate/formic acid (4/7/1, *v*/*v*/*v*) and ethyl acetate/water/formic acid (17/2/2, *v*/*v*/*v*) [21]. Twin trough chamber (CAMAG) was saturated for 20 min with the mobile phase before analysis. The chromatogram was developed upward until a distance of 80 mm and dried for 2 min in a stream of warm air. After 3 min of heating of the plates at 100 °C (TLC Plate Heater III, CAMAG), the spot pattern was developed by 0.5% solution of NTS in ethyl acetate (1 s, Chromatogram Immersion Device III, CAMAG). Distinction of the spots was performed after 5 min (plates were dried at room temperature in the fume hood), with 5% solution of PEG 4000 in dichloromethane for 1 s. Images were captured at 366 nm (CAMAG DigiStore 2 documentation system in conjunction with Reprostar 3 (CAMAG) controlled by the winCATS software (IZASA SCIENTIFIC, Madrid, Spain, Version 1.4.3.6336), four apertures with exposure time of 30 ms and frame of –2 mm [23], as tagged image file format (TIFF) files.

Image acquisition was performed by Image J processing program [24] using the procedure described previously [23]. Processing of images included the following steps: denoising (2 pixels median filter), baseline removal (none, background intensity between images were similar), normalization (scaling to sum of intensity), warping (correlation optimized warping (COW) algorithm with **P16** as target sample (PLS ToolBox, v.6.2.1, for MATLAB 7.12.0 (R2011a) [25], Eigenvector Research, Inc., Wenatchee, WA 98801), mean centering.

### 2.6. UHPLC–LTQ Orbitrap MS

An Accela liquid chromatography (LC) system connected to a linear ion trap–Orbitrap hybrid mass spectrometer (LTQ OrbitrapXL, Thermo Fisher Scientific) with heated electrospray ionization (HESI) was used for the identification of flavonoid glycosides in bee-pollen samples. Chromatographic separation and mass spectrometry parameters were same as previously reported by Guffa et al. [26].

### 2.7. Statistical Analysis

Principal component analysis (PCA) with a singular value decomposition algorithm (SVD) was performed using PLS ToolBox, v.6.2.1, provided by MATLAB 7.12.0 (R2011a). Kaiser Criterion was applied for optimal number of principal components (eigenvalues greater than 1 were cut-off).

## 3. Results and Discussion

### 3.1. Floral Origin of Bee-Pollen Loads

Palynological analysis (Table S1) for 22 samples [18] and for two additional samples identified 24 plant taxa and rust spores in bee-pollen. Eleven samples were monofloral, eight samples were bifloral and five samples were polyfloral [27].

### 3.2. HPTLC Fingerprint of Phenolics in Bee-Pollen Samples

Separation and assessment of all the ingredients of complex phenolic mixtures are very difficult to perform. Instead of targeted analysis of a metabolite, a characteristic chromatographic profile could be used for comparison which leads to sample recognition. In that sense, screening of phenolic mixtures of analyzed bee-pollen samples was performed by TLC analysis and obtained chromatograms observed as a unique multivariate fingerprint. A literature search revealed that no study on the identification of phenolics in bee-pollen by TLC has been published.

**Figure 1:**
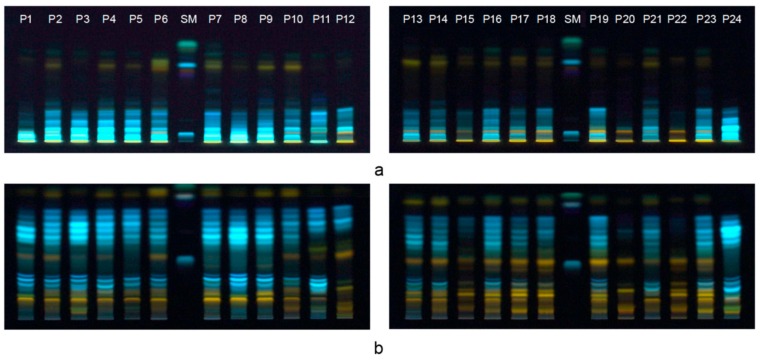
High-performance thin-layer chromatography (HPTLC) chromatograms of bee-pollen extracts, (**a**) chromatographic system (CS)1, (**b**) CS2. P—Pollen samples (Table S1); SM—standard mixture.

Due to the complexity of bee-pollen extracts, HPTLC systems were optimized in terms of the best resolution of different polarity phenolic compounds. Different chromatographic systems (CS1 and CS2) were used for the less polar fraction and for the medium and highly polar fraction, respectively. The standard mixture was simultaneously analyzed in both systems with substances obtained in the following ascending order of retention factor (*R*_F_) values: chlorogenic acid, gallic acid, myricetin, chrysin, caffeic acid, ferulic acid, naringenin, galangin. HPTLC chromatograms of bee-pollen extracts and the standard mixture are presented in Figure 1.

Chromatographic system 1 with low elution strength could not be used for the separation of a vast number of more polar compounds strongly adsorbed on silica gel (Figure 1a). However, non-polar flavonoids, such as flavonols, flavanons and isoflavonoids, showed specific patterns with orange and green colored zones which appear at higher *R*_F_ values. A different profile of these bands could be observed. CS2, used for the scanning of profiles of more polar compounds present in bee-pollen samples, gave chromatograms with good separation efficiency and low background noise. The applied mobile phase successfully separated medium and highly polar constituents (mainly the more polar phenolic acids such as chlorogenic, gallic and ellagic acids), and the different flavonoid aglycones (apigenin, quercetin, kaempferol, and their glycosides) [28,29]. Chromatographic profiles were rich in well defined, sharp zones which formed two dominant patterns, first with blue colored zones at *R*_F_ values between 0.6 and 0.8 which are present in all samples and could be considered as a characteristic feature of Serbian bee-pollen, and second with orange bands that could be attributed to very polar glycosides at lower *R*_F_ values, with profiles that depend on the samples and could be specific to certain botanical origins.

The chromatographic profiles gave basic information on the phenolic profile of bee-pollen samples, but could not provide enough detail to define the authenticity of bee-pollen and the parameters which could be used as markers of certain botanical origins. Further analysis was focused not on the particular sample but on the matrix of data consisting of all samples and each dot on the chromatograms.

**Figure 2:**
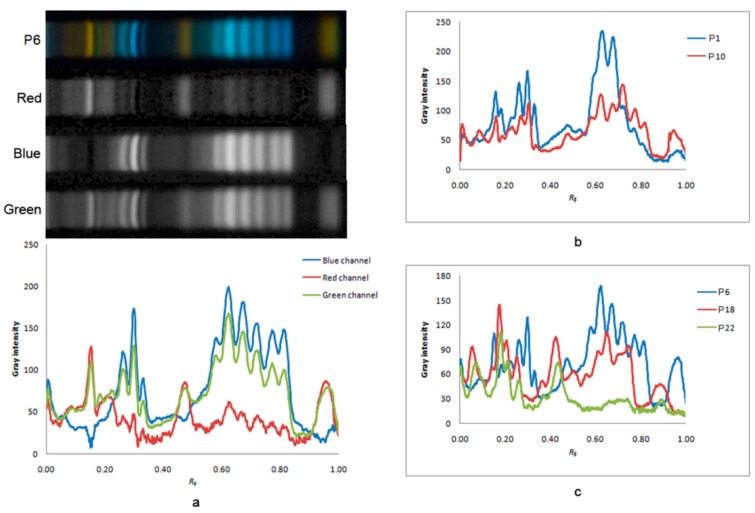
The line profile plots of chromatograms of (**a**) sample **P6** with a predomination of Apiaceae pollen type adjusted to three red/green/blue (RGB) channels, (**b**) samples **P1** and **P10**, respectively, adjusted to the green channel, (**c**) samples **P6**, **P18** and **P22**, respectively, adjusted to the green channel. *R_F_*: retention factor

In order to transfer results from TLC chromatograms to numerical values, multivariate image analysis was performed [30]. Due to the existence of different colored bands of phenolic compounds at 366 nm, in order to increase selectivity, images were split through red (R), green (G) and blue (B) channels. Red/green/blue (RGB) images were analyzed with Image J software and raw data were further processed by a pattern recognition method. The line profile plots of chromatograms for pollen samples of particular botanical origin, according to its RGB channels, are presented in Figure 2. Differences in profile plots of the same sample are the result of different color values of each point on the chromatographic profile depending on the channel applied. The line profile plots of the chromatogram corresponding to sample **P6** (predominantly Apiaceae pollen type) according to RGB channel are presented in Figure 2a. Visual review revealed that the green channel was the most informative for the analyzed pollen samples (Figure 2a). Also, phenolic line profile plots obtained for samples in which one pollen type predominates (samples **P1** and **P10** with a predomination of Brassicaceae and Fabaceae pollen type; **P6**, **P18** and **P22** with a predomination of Apiaceae, Ranunculaceae and *Sophora* pollen type, respectively) were shown to be notably different (Figure 2b,c).

Six data matrices for three channels and two chromatographic systems, each consisting of 24 samples and 526 variables (intensities of pixels along the length line), were subjected to PCA in order to mark compounds that are the most convenient for characterization of a particular pollen type. Pre-treatment of the data before chemometric evaluation enabled further classification (Figure S1, Supplementary Materials). The parameters of PCA models for six data sets revealed the best differentiation and classification results in case of the green channel of profiles obtained with CS1. Further, only this model will be discussed. The five-component model accounted for 93.04% of total variance (PC1—35.61% and PC2—33.70%).

**Figure 3:**
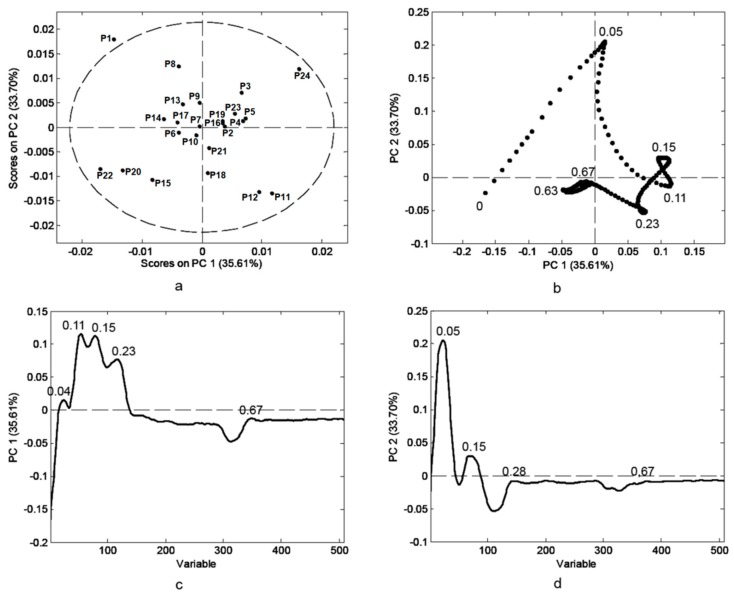
Principal component analysis (PCA) performed on data obtained from HPTLC phenolic profiles of bee-pollen samples, (**a**) mutual projections of factor scores, (**b**) loading plot for PC1 and PC2, (**c**,**d**) loadings for the PC1 and PC2, respectively.

According to the score plot (Figure 3a), two distinctive groups consisting of samples **P20** and **P22**, predominantly *Sophora* pollen, and samples **P11** and **P12**, predominantly Rosaceae and *Plantago* pollen, respectively, were classified in comparison to other samples where other pollen is more abundant. Samples **P15** (with rust spores), **P18** (with a high percentage of Ranunculaceae), and **P24** (with a high amount of Chenopodiaceae), are isolated on the graph. Pollen from Brassicaceae was predominant in five of the analyzed samples (**P1**, **P3**, **P8**, **P9**, and **P13**) and almost every other analyzed sample contained some percentage of it. Going along the PC1 axis from its negative end towards the positive values, the amount of this pollen type decreased. Samples **P1**, **P3**, **P8**, **P9**, and **P13**, with the highest amount of Brassicaceae pollen, are positioned in the left upper part of the graph. Sample **P3** was slightly separated from this group, which seems to be related to the notable amount of *Salix* pollen present which obviously has a high impact on the phenolic profile. Samples with predominantly Fabaceae pollen (samples **P2**, **P4**, **P5**, **P10**, **P16**, **P19**, and **P21**) formed a compact cluster positioned relatively close to the group with predominantly Brassicaceae pollen. Namely, all these samples contain Brassicaceae pollen in an amount of 3–25%. Apiaceae pollen seems to have an important influence on the phenolic profile, although it is present in small amounts. Samples **P6**, **P14** and **P17**, with 69%, 3–15%, and 28% of this pollen type, respectively, are grouped very close together and close to clusters with predominantly Brassicaceae and Fabaceae pollen, and therefore probably contain some identical phenolics. Sample **P23**, which contains predominantly *Robinia* pollen, was positioned within the group that has predominantly Fabaceae pollen, suggesting that it has a similar phenolic content, which is in line with the fact that false locust (*Robinia*) belongs to the Fabaceae plant family. There are a certain number of scientific works that deal with determining the profile of a variety of chemical compounds in order to define the botanical origin of bee-pollen [7,31]. Since the bee-pollen samples investigated in this study present a mixture of more than ten different pollen types, differentiation and classification based on chemical profile and distinct separation among the groups are difficult to perform, particularly when observing only one class of compounds and not combining several groups of parameters. Similar results were obtained based on NIR spectra of bee-pollen samples collected from twelve different Brazilian states [32], while Castiglioni et al. suggest the possibility of using IR measurements for the classification of some Italian bee-pollen samples according their botanical origin [31]. The loading plots (Figure 3b–d) reveal that the zones with *R*_F_ values of 0.04, 0.11, 0.15, and 0.23 are parameters that showed the most positive impact on PC1 direction and can differentiate pollen samples according to the botanical type. Zones with *R*_F_ values of 0.05 and 0.15 significantly affect the PC2 in a positive manner, while zones with a negative influence on the second component have *R*_F_ values of 0.23 and 0.63.

### 3.3. Identification of Flavonoid Glycosides by UHPLC–LTQ Orbitrap MS

**Figure 4:**
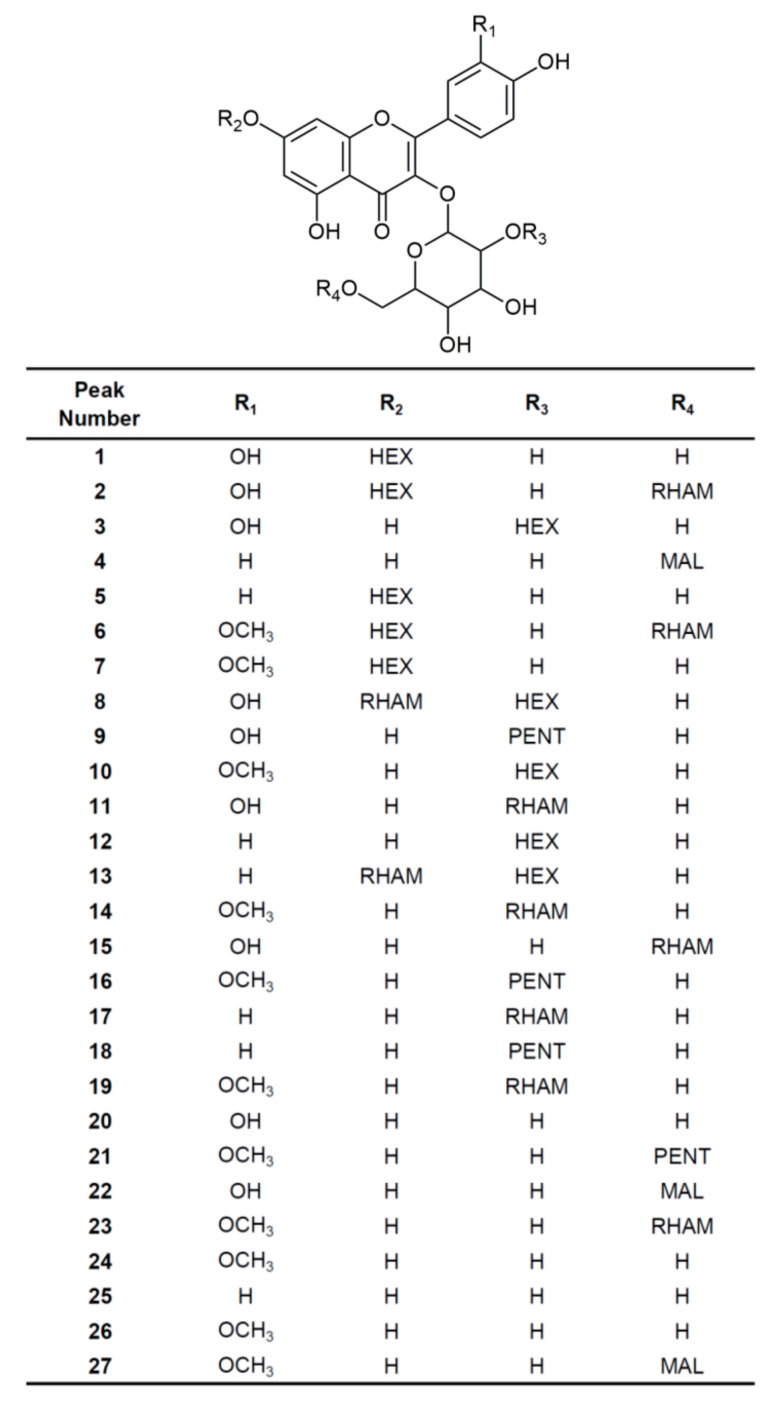
Chemical structures of different flavonol glycosides found in the bee-pollen collected in Serbia (R_1–4_ = HEX—hexosyl, RHAM—rhamnosyl, PENT—pentosyl or MAL—malonyl.).

The qualitative profile of flavonoid glycosides with mono-, di- and trisaccharide moieties present in bee-pollen samples was determined using an UHPLC system coupled to an LTQ Orbitrap mass analyzer. Their characteristics (mass spectra, accurate mass, MS/MS fragmentation pattern and characteristic retention time) were used for their identification. We also used available standards as another confirmation for these compounds. It was found that all of the identified compounds are from the same group of flavonols and all of them were marked as 3-*O*-glycosides of quercetin, isorhamnetin, and kaempferol. The exact mass search and the study of the MS/MS fragmentation described in the literature enabled us to identify 27 flavonol glycosides (Figure 4 and Table 1).

The obtained data on the retention times (t*_R_*, min) and MS parameters for the identified glycosides are summarized in Table 1. The presence of each identified peak (glycoside) from all bee-pollen samples is shown in Table S2 (Supplementary Material). Tentative identification of flavonol glycosides was done using the MS fragmentation data available in the literature [5,6,7,8,33]. The nature of interglycosidic linkages (1→2 or 1→6) for the identified flavonol glycosides was proposed according to Ferreres et al. [11].

MS/MS fragmentation of deprotonated molecular ion [M−H]^−^ at *m*/*z* 447 (compound **25**) produced the fragment ion Y_0_^−^ at *m*/*z* 285 ([M−H−162]^−^) together with the formation of the radical anion [Y_0_-H]^−^ at *m*/*z* 284. The observed fragment *m*/*z* 327 originates from the ^0,2^X^−^ scission of the glycoside [34], indicating that the structure of compound **25** is kaempferol 3-*O*-glucoside or astragalin, which is confirmed by the standard.

Furthermore, identification of two derivatives of isorhamnetin 3-*O*-hexoside (compounds **24** and **26**) with *m*/*z* 477 was established from the presence of the Y_0_^−^ ion and radical anion [Y_0_−H]^−^ at *m*/*z* 315 and 314, respectively. Fragment ion at *m*/*z* 449 present in compound **26** originated from loss of small neutral molecule CO [M−H−28]^−^.

Compound **20** exhibited a deprotonated molecular ion [M−H]^−^ at *m*/*z* 463. The MS/MS spectrum in the negative ionization mode showed fragments Y_0_^−^ at *m*/*z* 301 ([M−H−162]^−^) and a radical anion [Y_0_−H]^−⋅^ at *m*/*z* 300, suggesting the loss of the hexosyl moiety and quercetin as aglycone. The proposed identification of compound **20** is quercetin 3-*O*-galactoside or hyperoside, which was confirmed by the standard. A similar fragmentation pattern for compounds **4** (*m*/*z* 533), **22** (*m*/*z* 549), and **27** (*m*/*z* 563) was observed with a specific fragment ion at [M−H−44]^−^, indicating the presence of a carboxylic group (malonyl moiety) [33]. Further examination of mass spectra and molecular formulas of these compounds allowed us to identify these glycosides as kaempferol 3-*O*-(6”-*O*-malonyl)hexoside, quercetin 3-*O*-(6”-*O*-malonyl)hexoside and isorhamnetin 3-*O*-(6”-*O*-malonyl)hexoside, respectively.

In the negative ion MS/MS spectra of compounds **11** (*m*/*z* 609), **14** (*m*/*z* 623), **15** (*m*/*z* 609), **17** (*m*/*z* 593), **19** (*m*/*z* 623), and **23** (*m*/*z* 623), the Y_0_^−^ [M−H−308]^−^ fragment originating from the losses of the rhamnosyl-hexoside moiety was observed. Ion product spectra of these compounds also yielded radical ions [Y_0_–H]^−⋅^ with higher intensity which are characteristic for 3-*O*-glycosides [35]. The relative abundance of the radical [Y_0_–H]^−⋅^ is higher than the deprotonated Y_0_^−^ ion for compounds **11***,* **14**, **17**, and **19**. In addition, fragment ions that originate from ^0,2^X^−^ scission of the terminal rhamnose residue [M−H−120]^−^ together with formation of the [M−H−164]^−^ by loss of rhamnose molecule, are also present in the mass spectra of these compounds and not observed for compounds **15** and **23**. This means that the interglycosidic linkage in compounds **11**, **14**, **17***,* and **19** was the 1→2 type, while in compounds **15** and **23**, it was the 1→6 interglycosidic linkage. [36]. Based on these considerations, we could identify compounds **11**, **14**, **17**, and **19** as quercetin 3-*O*-(2”-*O*-rhamnosyl)hexoside; isorhamnetin 3-*O*-(2”-*O*-rhamnosyl)hexoside, isomer 1; kaempferol 3-*O*-(2”-*O*-rhamnosyl)hexoside; and isorhamnetin 3-*O*-(2”-*O*-rhamnosyl)hexoside, isomer 2, respectively. The proposed fragmentation pathways of compounds **14** and **19** are depicted in Figure S2. Compound **23** was tentatively identified as isorhamnetin 3-*O*-(6”-*O*-rhamnosyl)hexoside and compound **15** was marked as quercetin 3-*O*-(6”-*O*-rhamnosyl)glucoside or rutin (also confirmed by the standard).

MS/MS spectrum of compounds **3** (*m*/*z* 625), **10** (*m*/*z* 639), and **12** (*m*/*z* 609) in the negative ionization mode, showed fragment Y_0_^−^ and the radical anion [Y_0_–H]^−^, suggesting the loss of two hexose units. Fragments that originated by losses of the hexosyl moiety at ([M−H−162]^−^) and hexosyl and H_2_O at ([M−H−162−18]^−^) were characteristic for sugar units linked one to another (and not directly to the aglycone) were also presented as well as fragment ions that originate from ^0,2^X^−^ scission of the terminal hexose residue [M−H−120]^−^. This information allows us to determine that compounds **3**, **10**, and **12** are 3-*O*-(2”-*O*-hexosyl)hexosides of quercetin, isorhamnetin, and kaempferol, respectively [37].

There are similar fragmentation patterns for compounds **9** (*m*/*z* 595), **16** (*m*/*z* 609), and **18** (*m*/*z* 579). The presence of Y_0_^−^ fragment ions at *m*/*z* 301, *m*/*z* 315 and *m*/*z* 285 indicated that in these compounds, the aglycones were quercetin, kaempferol and isorhamnetin, and it also suggested successive losses of pentosyl (132 Da) and hexosyl (162 Da) moieties. According to presence of characteristic fragments ([M−H−120]^−^, [M−H−132]^−^, and [M−H−132−18]^−^) in MS/MS spectra, these compounds were marked as 3-*O*-(2”-*O*-pentosyl)hexosides of quercetin, isorhamnetin, and kaempferol, respectively.

Compound **21** (*m*/*z* 609) is an isomer of compound **16**. In its mass spectra, only the Y_0_^−^ ion at *m*/*z* 315 was observed, indicating a 1→6 interglycosidic linkage between sugars, so we determined compound **21** was isorhamnetin 3-*O*-(6”-*O*-pentosyl)hexoside.

In the negative ionization mode, MS/MS spectra of compounds **1** (*m*/*z* 625), **5** (*m*/*z* 609), and **7** (*m*/*z* 639) showed high intensity of MS/MS fragments at [M−H−162]^−^ (*m*/*z* 463, 447, and 477, respectively) corresponding to loss of hexose in the C−3 or C−7 position. Based on these findings, the proposed identification for these compounds was quercetin 3,7-di-*O*-hexoside, kaempferol 3,7-di-*O*-hexoside, and isorhamnetin 3,7-di-*O*-hexoside, respectively [11].

Compounds **2** (*m*/*z* 771), **6** (*m*/*z* 785), **8** (*m*/*z* 771), and **13** (*m*/*z* 755) are triglycosides. MS/MS spectra of these compounds exhibited fragment [M−H−146−162−162]^−^ which correspond to aglycones and the loss of two hexosyl and one deoxyhexosyl (rhamnosyl) unit. Characteristic fragments in the MS/MS spectra of compounds **8** and **13** were at *m*/*z* 625 and 609, indicating the loss of a rhamnosyl moiety from the hydroxyl group at the C−7 position [38]. Characteristic fragments [M−H−146−162−18]^−^ at *m*/*z* 445 and 429 were also present. Therefore, the proposed structures of compounds **8** and **13** were 3-*O*-(2”-*O*-hexosyl)hexoside-7-*O*-rhamnosides of quercetin and kaempferol, respectively [39,40]. Compound **2** is an isomer of compound **8**, but its fragmentation pattern, with Y_0_^−^ fragment ions at *m*/*z* 301 (deprotonated quercetin) and at *m*/*z* 609 ([M−H−162]^−^), corresponds to compound **15** with one more hexose unit. Therefore, compound **2** was marked as quercetin 3-*O*-(6”-*O*-rhamnosyl)hexoside-7-*O*-hexoside. The proposed structure of compound **6** was isorhamnetin 3-*O*-(6”-*O*-rhamnosyl)hexoside-7-*O*-hexoside, due to its similar spectral characteristics as compound **2** [41].

Analyzed bee-pollen samples are mixtures of more than ten different floral pollen types so the twenty-seven identified flavonol glycosides was expected. Each of the analyzed bee-pollen samples contains over fourteen flavonol glycosides, except for sample **P20** (with a predomination of the *Sophora* pollen type) which has twelve of these compounds. The largest number of flavonol glycosides (total of 25) has been identified in the sample **P11** (with a predomination of the Rosaceae pollen type). Several compounds were identified in bee-pollen samples for the first time, including compounds **2**, **6**, **7**, **8**, **9**, **13**, **16**, and **21**. Compounds **3**, **4**, **17** and **20** are present in all bee-pollen samples. Their quantification could help with the identification of floral pollen types by using flavonol glycosides as markers to classify pollen from different plant species [42], but this was not conducted due to the lack of appropriate standards. The characterization of bee-pollen, in terms of their constituent pollens, would be easier if bee-pollen pellets were manually selected on the basis of color and size. It has been proven that certain bee-pollen pellet types contain pollen from a single floral source [3]. However, in our research, we opted for a mixture because the bee-pollen in this form is sent to the market. Separation into families would otherwise be unprofitable for beekeepers.

### 3.4. The Total Phenolic Content of Bee-Pollen Samples

Bee-pollen samples were characterized with TPC values ranging between 5.60 and 30.24 mg of gallic acid per gram of pollen (Table S3, Supplementary Material). The highest level of total phenolics was found in sample **P15** (with the most dominant rust spores) and the lowest level was determined in sample **P5** (with a predomination of the Fabaceae pollen type). Generally, all samples with Fabaceae as the predominant pollen type showed lower TPC values. In addition, samples with a predomination of the *Sophora* pollen type also had low levels of total phenols. The range of phenolic compound content was wider in relation to the previously published results. Mărghitaş et al. [43] found TPC values between 4.4 mg and 16.4 mg GAE/g in twelve bee-pollen samples from the Transylvania area of Romania, while in ten bee-pollen samples from Turkey, TPC values ranged from 5.09 mg to 17.46 mg GAE/g [44]. Additionally, the maximal concentration of phenolic compounds found in our study was higher than those reported for bee-pollen from five Portuguese natural parks, which were between 10.5 mg and 16.8 mg GAE/g of pollen [16]. The results of Le Blanc et al. [17] for bee-pollen from the Sonoran Desert, and Pascoal et al. [45], who analyzed bee-pollen from Portugal and Spain, were slightly superior due to phenolic contents between 15.91 mg and 34.85 mg GAE/g and 18.54 mg and 32.15 mg GAE/g, respectively. We assumed that these differences between TPC could be ascribed to different botanical and geographical origins of the analyzed bee-pollen samples.

## 4. Conclusions

A comprehensive study of the phenolic compounds in bee-pollen samples from Serbia was conducted. HPTLC fingerprinting confirmed the grouping of samples caused by specific phenolic compositions of pollens of different botanical origin. A UHPLC system coupled to an LTQ Orbitrap mass analyzer allowed for the identification of twenty-seven flavonol glycosides. Eight of them were identified in bee-pollen samples for the first time. Determination of species-specific phenolic profiles is needed for the precise characterization of samples in terms of constituent pollens. We assume that this could be achieved by the quantification of certain flavonol glycosides and sorting bee-pollen pellets on the basis of color and size. Analyzed bee-pollen samples have high amounts of phenolic compounds, which may have therapeutic potential. The findings presented here corroborate the relevance of bee-pollen from Serbia as a healthy addition to the human diet.

## Figures and Tables

**Table 1:** Peak numbers, retention times (*t*_R_), molecular formulas, calculated and exact masses, mean mass accuracy (ppm), and MS/MS fragments of flavonol glycosides identified in bee-pollen samples.

Peak Number	t*_R_*, min	Molecular Formula, [M–H]^−^	Calculated Mass, [M–H]^−^	Exact Mass, [M–H]^−^	Δ, ppm	MS/MS Fragmentation (%)	Identification
**1**	5.30	C_27_H_29_O_17_^−^	625.14102	625.14233	2.10	505(5), 463(100), 462(20), 343(5), 301(30)	Quercetin 3,7-di-*O*-hexoside
**2**	5.74	C_33_H_39_O_21_^−^	771.19893	771.20038	1.88	609(100), 463(10), 301(5)	Quercetin 3-*O*-(6″-*O*-rhamnosyl)hexoside-7-*O*-hexoside ^b^
**3**	5.85	C_27_H_29_O_17_^−^	625.14102	625.14172	1.12	505(15), 463(15), 445(50), 343 (10), 301(50), 300(100), 271(20), 255(10)	Quercetin 3-*O*-(2″-*O*-hexosyl)hexoside
**4**	5.89	C_24_H_21_O_14_^−^	533.09368	533.09454	1.61	489(100), 285(5)	Kaempferol 3-*O*-(6″-*O*-malonyl)hexoside
**5**	5.90	C_27_H_29_O_16_^−^	609.14611	609.14636	0.41	581(10), 489(10), 447(70), 446(50), 327(5), 285(100)	Kaempferol 3,7-di-*O*-hexoside
**6**	5.91	C_34_H_41_O_21_^−^	785.21458	785.21509	0.65	623(100), 477(10), 315(5)	Isorhamnetin 3-*O*-(6″-*O*-rhamnosyl)hexoside-7-*O*-hexoside^b^
**7**	5.96	C_28_H_31_O_17_^−^	639.15667	639.15717	0.78	477(40), 476(20), 357(5), 315(100), 300(10)	Isorhamnetin 3,7-di-*O*-hexoside ^b^
**8**	5.98	C_33_H_39_O_21_^−^	771.19893	771.20056	2.11	753(10), 625(100), 609(30), 591(40), 573(20), 445(10), 427(10), 409(10), 301(45), 300(50)	Quercetin 3-*O*-(2″-*O*-hexosyl)hexoside-7-*O*-rhamnoside^b^
**9**	6.00	C_26_H_27_O_16_^−^	595.13046	595.13098	0.87	475(10), 463(10), 445(15), 301(45), 300(100), 271(20), 255(10)	Quercetin 3-*O*-(2″-*O*-pentosyl)hexoside ^b^
**10**	6.02	C_28_H_31_O_17_^−^	639.15667	639.15784	1.83	624(20), 519(15), 491(10), 477(20), 459(65), 444(30), 315(100), 314(50), 300(50), 299(60)	Isorhamnetin 3-*O*-(2″-*O*-hexosyl)hexoside
**11**	6.04	C_27_H_29_O_16_^−^	609.14611	609.14642	0.51	489(15), 463(10), 445(20), 429(10), 343(5), 301(20), 300(100), 285(5), 271(15), 255(10)	Quercetin 3-*O*-(2″-*O*-rhamnosyl)hexoside
**12**	6.07	C_27_H_29_O_16_^−^	609.14611	609.14697	1.41	489(5), 447(10), 429(100), 327(10), 285(90), 284(80), 255(15)	Kaempferol 3-*O*-(2″-*O*-hexosyl)hexoside
**13**	6.19	C_33_H_39_O_20_^−^	755.20402	755.20471	0.91	609(100), 593(90), 575(85), 429(20), 285(90), 284(70), 255(25)	Kaempferol 3-*O*-(2″-*O*-hexosyl)hexoside-7-*O*-rhamnoside ^b^
**14**	6.22	C_28_H_31_O_16_^−^	623.16176	623.16211	0.56	608(10), 503(10), 477(10), 459(30), 444(10), 327(10), 315(15), 314(100), 300(10), 299(65)	Isorhamnetin 3-*O*-(2″-*O*-rhamnosyl)hexoside isomer 1
**15**	6.24	C_27_H_29_O_16_^−^	609.14611	609.14679	1.12	343(5), 301(100), 300(20), 271(10), 255(5)	Quercetin 3-*O*-(6″-*O*-rhamnosyl)glucoside (Rutin) ^a^
**16**	6.26	C_27_H_29_O_16_^−^	609.14611	609.14716	1.72	577(15), 489(5), 477(5), 459(50), 357(10), 315(100), 314(90), 300(20), 299(30)	Isorhamnetin 3-*O*-(2″-*O*-pentosyl)hexoside ^b^
**17**	6.28	C_27_H_29_O_15_^−^	593.15119	593.15161	0.71	473(5), 447(10), 429(50), 327(5), 285(40), 284(100), 255(20)	Kaempferol 3-*O*-(2″-*O*-rhamnosyl)hexoside
**18**	6.30	C_26_H_27_O_15_^−^	579.13554	579.13678	2.14	459(5), 447(20), 429(60), 327(10), 285(75), 284(100), 255(30)	Kaempferol 3-*O*-(2″-*O*-pentosyl)hexoside
**19**	6.34	C_28_H_31_O_16_^−^	623.16176	623.16248	1.16	591(5), 503(10), 477(10), 459(25), 357(5), 315(35), 314(100), 300(10), 299(25)	Isorhamnetin 3-*O*-(2″-*O*-rhamnosyl)hexoside isomer 2
**20**	6.39	C_21_H_19_O_12_^−^	463.08820	463.08951	2.83	301(100), 300(20)	Quercetin 3-*O*-galactoside (Hyperoside) ^a^
**21**	6.43	C_27_H_29_O_16_^−^	609.14611	609.14697	1.41	315(100), 300(20), 271(10), 255(5)	Isorhamnetin 3-*O*-(6″-*O*-pentosyl)hexoside ^b^
**22**	6.54	C_24_H_21_O_15_^−^	549.08859	549.08923	1.17	505(100), 301(5)	Quercetin 3-*O*-(6″-*O*-malonyl)hexoside
**23**	6.57	C_28_H_31_O_16_^−^	623.16176	623.16272	1.54	315(100), 314(10), 300(20), 271(10), 255(5)	Isorhamnetin 3-*O*-(6″-*O*-rhamnosyl)hexoside
**24**	6.65	C_22_H_21_O_12_^−^	477.10385	477.10477	1.93	315(100), 314(20)	Isorhamnetin 3-*O*-hexoside isomer 1
**25**	6.70	C_21_H_19_O_11_^−^	447.09329	447.09424	2.12	327(20), 285(80), 284(100), 255(10)	Kaempferol 3-*O*-glucoside (Astragalin) ^a^
**26**	6.78	C_22_H_21_O_12_^−^	477.10385	477.10437	1.09	449(5), 357(15), 315(30), 314(100)	Isorhamnetin 3-*O*-hexoside isomer 2
**27**	6.86	C_25_H_23_O_15_^−^	563.10424	563.10486	1.10	519(100), 315(5)	Isorhamnetin 3-*O*-(6″-*O*-malonyl)hexoside

^a^ Confirmed using available standards; all other compounds were identified based on MS/MS data. ^b^ Identified for the first time in bee-pollen.

## Supplementary Materials

The following are available online at https://www.mdpi.com/2218-273X/9/12/783/s1, Table S1: Frequency classes of identified pollen types and interpretation of floral origin from results of pollen analysis published in Kostić et al., 2015 [18], Table S2: Presence of each identified glycoside in bee-pollen samples, Table S3: Total phenolic content (TPC) of bee pollen samples, Figure S1: Raw data (a) versus preprocessed data (b) of HPTLC profiles, Figure S2: Proposed fragmentation pathway of compounds 14 and 19 (*m*/*z* 623).
