# Supplementary material for: Phenolic Composition Influences the Health-Promoting Potential of Bee-Pollen
Source: Biomolecules. 2019 Nov 26;9(12):783. doi: 10.3390/biom9120783 (PMC6995608; doi:10.3390/biom9120783)
Supplement: Supplementary file 1 [file biomolecules-09-00783-s001.pdf]

Article - Supplementary material

Phenolic Composition Influences the Health-Promoting Potential of Bee-Pollen

Mirjana Mosić [1], Jelena Trifković [1], Irena Vovk [2], Uroš Gašić [3,*], Živoslav Tešić [1], Branko Šikoparija [4] and Dušanka Milojković-Opsenica [1,*]

[1] University of Belgrade—Faculty of Chemistry P.O. Box 51, 11158 Belgrade, Serbia
[2] Department of Food Chemistry, National Institute of Chemistry, Hajdrihova 19, SI-1000 Ljubljana, Slovenia
[3] Institute for Biological Research "Siniša Stanković"—National Institute of Republic of Serbia, University of Belgrade, Bulevar despota Stefana 142, 11060 Belgrade, Serbia
[4] BioSense Institute—Research Institute for Information Technologies in Biosystems, University of Novi Sad, 21000 Novi Sad
* Correspondence: uros.gasic@ibiss.bg.ac.rs; dusankam@chem.bg.ac.rs (D.M.-O.); Tel.: +381112078385 (U.G.) Tel.: +381113336766 (D.M.-O.)

Content

Table S1. Frequency classes [27] of identified pollen types and interpretation of floral origin from results of pollen analysis published in [18].

Table S2. Presence of each identified glycoside in bee-pollen samples.

Table S3. Total phenolic content (TPC) of bee-pollen samples.

Figure S1. Raw data (a) versus preprocessed data (b) of HPTLC profiles.

Figure S2. Proposed fragmentation pathway of compounds **14** and **19** (*m*/*z* 623).

Table S1. Frequency classes [23] of identified pollen types and interpretation of floral origin from results of pollen analysis published in Kostić *et al.*, [16].

Sample No.	Sample No. from [16]	Total Pollen Types	Very Frequent (>85%)	Frequent (46–85%)	Accompanying (16–45%)	Important Isolated (3–15%)	Floral Origin
**P1**	2	5	Brassicaceae (93%)		-	Moraceae	MONOFLORAL (Brassicaceae)
**P2**	3	25	-	Fabaceae (48%)	Brassicaceae (19%)	Ranunculaceae, *Vitis*	BIFLORAL (Fabaceae, Brassicacea)
**P3**	5	14	-	-	Brassicaceae (45%), *Salix* (35%)	Rosaceae, *Vitis*	BIFLORAL (Brassicaceae, Salix)
**P4**	8	15	-	Fabaceae (72%)	-	Brassicaceae, *Sophora*	MONOFLORAL (Fabaceae)
**P5**	9	15	-	Fabaceae (50%)	-	Brassicaceae, Rosaceae, *Salix*, *Vitis*	MONOFLORAL (Fabaceae)
**P6**	10	12	-	Apiaceae (69%)	-	-	MONOFLORAL (Apiaceae)
**P7**	11	18	-	-	Brassicaceae (31%), Fabaceae (18%), Moraceae (18%)	Lamiaceae, *Salix*, *Tilia*	POLYFLORAL
**P8**	12	12	-	Brassicaceae (76%)	-	*Salix*, Rosaceae, *Sambucus*, Apiaceae	MONOFLORAL (Brassicaceae)
**P9**	13	15	-	Brassicaceae (53%)	-	Apiaceae, Asteraceae, Fabaceae, Ranunculaceae	MONOFLORAL (Brassicaceae)
**P10**	14	13	-	Fabaceae (81%)	-	Brassicaceae	MONOFLORAL (Fabaceae)
**P11**	15	22	-	-	Rosaceae (42%), Fabaceae (23%)	Asteraceae, *Plantago*	BIFLORAL (Rosaceae, Fabaceae)
**P12**	16	10	-	-	*Plantago* (35%), Fabaceae (19%), *Ambrosia* (19%), Asteraceae (18%)	*Zea mays*	POLYFLORAL
**P13**	17	18	-	-	Brassicaceae (34%), Fabaceae (28%)	*Helianthus*, Ranunculaceae, *Tilia*	BIFLORAL (Brassicaceae, Fabaceae)
**P14**	18	17	-	-	Asteraceae (31%), *Ambrosia* (18%)	Asteraceae, Apiaceae, *Artemisia*, *Helianthus*, Brassicaceae, Chenopodiaceae, Ranunculaceae,	POLYFLORAL
**P15**	19	23	-	-	Asteraceae (18%)	Rust spores, Asteraceae, *Helianthus*, *Carduus*, Brassicaceae, Fabaceae, *Plantago*, Poaceae, *Zea mays*, Ranunculaceae, Rosaceae,	POLYFLORAL
**P16**	20	22	-	-	Fabaceae (34%), Brassicaceae (25%)	*Artemisia*, *Ambrosia*, Ranunculaceae, *Vitis*	BIFLORAL (Fabaceae, Brassicaceae)
**P17**[1]	-	17	-	-	Ranunculaceae (33%), Apiaceae (28%)	-	BIFLORAL (Ranunculaceae,

							Apiaceae)
**P18**	21	18	-	Ranunculaceae (76%)	-	Asteraceae, *Artemisia*, *Helianthus*	MONOFLORAL (Ranunculaceae)
**P19**	22	17	-	Fabaceae (57%)	-	Brassicaceae, *Ambrosia*, *Helianthus*, *Vitis*	MONOFLORAL (Fabaceae)
**P20**	23	7	-	-	*Sophora* (42%)	Asteraceae, Cannabaceae, Brassicaceae	MONOFLORAL (*Sophora*)
**P21**	24	18	-	Fabaceae (78%)	-	Brassicaceae	MONOFLORAL (Fabaceae)
**P22**	25	12	-	*Sophora* (46%)	*Helianthus* (21%)	Asteraceae, Chenopodiaceae, *Zea mays*. Ranunculaceae	BIFLORAL (*Sophora*, *Helianthus*)
**P23**	26	19	-	-	Ranunculaceae (24%), *Robinia* (22%)	Asteraceae, *Artemisia*, *Ambrosia*, Chenopodiaceae, Brassicaceae, *Cornus*	POLYFLORAL
**P24**[1]	-	14	-	-	Apiaceae (38%), Chenopodiaceae (20%)	Asteraceae, *Artemisia*, Brassicaceae, Moraceae, Poaceae, *Zea mays*, unidentified-large reticulate	BIFLORAL (Apiaceae, Chenopodiaceae)

[1]Samples **P17** and **P24** were not analysed in Kostić *at al*., 2015 [16].

Table S2. Presence of each identified glycoside in bee-pollen samples.

Peak N°.	Flavonol Glycosides	Bee-pollen samples																							
		P1	P2	P3	P4	P5	P6	P7	P8	P9	P10	P11	P12	P13	P14	P15	P16	P17	P18	P19	P20	P21	P22	P23	P24
1	Quercetin 3,7-di-*O*-hexoside	–	–	–	–	–	+	–	–	–	+	–	+	–	–	+	–	–	+	+	+	+	+	–	+
2	Quercetin 3-*O*-(6"-*O*-rhamnosyl)hexoside-7-*O*-hexoside	+	+	–	+	+	+	+	+	–	+	+	+	+	+	+	+	+	+	+	+	+	+	+	+
3	Quercetin 3-*O*-(2"-*O*-hexosyl)hexoside	+	+	+	+	+	+	+	+	+	+	+	+	+	+	+	+	+	+	+	+	+	+	+	+
4	Kaempferol 3-*O*-(6"-*O*-malonyl)hexoside	+	+	+	+	+	+	+	+	+	+	+	+	+	+	+	+	+	+	+	+	+	+	+	+
5	Kaempferol 3,7-di-*O*-hexoside	+	+	+	+	+	–	+	+	+	+	+	–	+	+	+	+	+	–	+	–	–	–	+	–
6	Isorhamnetin 3-*O*-(6"-*O*-rhamnosyl)hexoside-7-*O*-hexoside	–	+	+	+	–	+	+	–	–	+	+	+	+	+	+	–	+	+	+	+	+	+	+	+
7	Isorhamnetin 3,7-di-*O*-hexoside	–	–	–	–	–	+	–	–	+	+	+	+	–	+	+	–	–	+	+	+	+	+	+	+
8	Quercetin 3-*O*-(2"-*O*-hexosyl)hexoside-7-*O*-rhamnoside	+	+	+	+	+	+	+	+	+	+	+	–	+	+	–	+	+	+	+	–	+	+	+	+
9	Quercetin 3-*O*-(2"-*O*-pentosyl)hexoside	+	+	+	+	+	+	+	+	+	+	+	+	+	+	+	+	+	+	+	+	+	+	+	+
10	Isorhamnetin 3-*O*-(2"-*O*-hexosyl)hexoside	+	+	+	+	+	+	+	+	+	+	+	–	+	–	+	+	+	+	+	–	+	–	+	–
11	Quercetin 3-*O*-(2"-*O*-rhamnosyl)hexoside	–	+	+	+	+	+	–	–	+	+	+	+	–	–	–	–	+	+	+	+	+	+	+	+
12	Kaempferol 3-*O*-(2"-*O*-hexosyl)hexoside	+	+	+	+	+	+	+	+	+	+	+	–	+	+	+	+	+	–	+	–	+	+	+	+
13	Kaempferol 3-*O*-(2"-*O*-hexosyl)hexoside-7-*O*-rhamnoside	+	+	+	+	+	+	+	+	+	+	+	+	+	+	–	+	–	–	+	–	–	–	–	+
14	Isorhamnetin 3-*O*-(2"-*O*-rhamnosyl)hexoside isomer 1	+	+	+	+	+	+	+	+	+	+	+	–	+	+	+	+	+	+	+	–	+	–	+	–
15	Quercetin 3-*O*-(6"-*O*-rhamnosyl)hexoside	–	–	–	–	–	–	–	–	–	–	–	–	–	–	–	–	–	–	–	–	+	–	+	–
16	Isorhamnetin 3-*O*-(2"-*O*-pentosyl)hexoside	–	–	–	–	–	+	–	–	–	–	+	+	–	–	+	+	–	+	+	+	–	–	+	+
17	Kaempferol 3-*O*-(2"-*O*-rhamnosyl)hexoside	+	+	+	+	+	+	+	+	+	+	+	+	+	+	+	+	+	+	+	+	+	+	+	+
18	Kaempferol 3-*O*-(2"-*O*-pentosyl)hexoside	+	+	+	+	+	+	+	+	+	+	+	–	+	+	+	+	+	+	+	–	+	+	+	+
19	Isorhamnetin 3-*O*-(2"-*O*-rhamnosyl)hexoside isomer 2	–	+	+	+	+	+	–	+	+	+	+	+	–	+	+	–	+	+	+	+	+	+	+	+
20	Quercetin 3-*O*-hexoside	+	+	+	+	+	+	+	+	+	+	+	+	+	+	+	+	+	+	+	+	+	+	+	+
21	Isorhamnetin 3-*O*-(6"-*O*-pentosyl)hexoside	–	–	+	+	+	–	–	–	–	–	+	–	–	–	–	–	–	–	–	–	–	–	–	–
22	Quercetin 3-*O*-(6"-*O*-malonyl)hexoside	+	+	+	+	–	+	–	+	+	+	+	+	+	+	+	–	+	+	+	+	+	+	+	+
23	Isorhamnetin 3-*O*-(6"-*O*-rhamnosyl)hexoside	–	–	–	–	–	+	+	–	–	+	+	+	–	+	+	–	+	+	+	+	+	+	+	+
24	Isorhamnetin 3-*O*-hexoside isomer 1	+	+	+	+	+	–	+	+	+	+	+	–	+	–	–	+	–	–	–	–	+	–	–	–
25	Kaempferol 3-*O*-hexoside	+	+	+	+	+	+	+	+	+	+	+	+	+	+	+	+	+	+	+	–	+	+	+	+
26	Isorhamnetin 3-*O*-hexoside isomer 2	–	+	+	+	–	+	+	+	–	+	+	+	+	+	+	–	+	+	+	+	+	+	+	+
27	Isorhamnetin 3-*O*-(6"-*O*-malonyl)hexoside	+	+	+	+	+	+	+	+	+	+	+	–	+	–	–	+	+	–	–	–	–	–	–	–

+ stands for detected: − stands for not detected.

Table S3. Total phenolic content (TPC) of the bee-pollen samples.

Sample No	TPC mg GAE/g
**P1**	15.504 ± 0.146
**P2**	10.925 ± 0.136
**P3**	13.607 ± 0.111
**P4**	8.303 ± 0.059
**P5**	5.600 ± 0.351
**P6**	13.563 ± 0.176
**P7**	23.004 ± 0.351
**P8**	17.983 ± 0.381
**P9**	16.572 ± 0.117
**P10**	8.581 ± 0.088
**P11**	11.819 ± 0.171
**P12**	9.607 ± 0.029
**P13**	12.146 ± 0.039
**P14**	10.990 ± 0.069
**P15**	30.244 ± 0.049
**P16**	11.928 ± 0.098
**P17**	14.286 ± 0.293
**P18**	11.872 ± 0.078
**P19**	13.132 ± 0.234
**P20**	6.542 ± 0.293
**P21**	11.402 ± 0.084
**P22**	5.696 ± 0.107
**P23**	13.004 ± 0.224
**P24**	17.793 ± 0.127

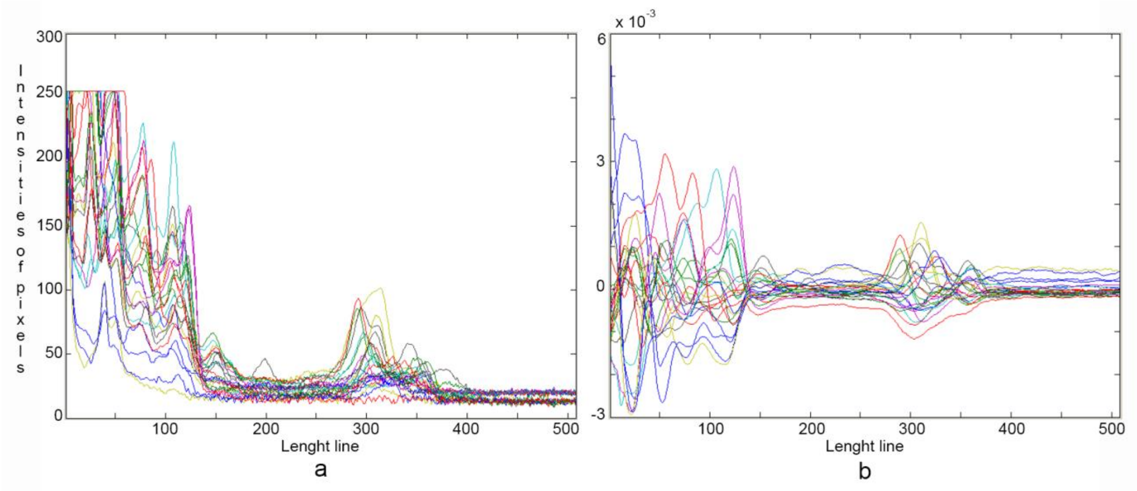

Figure S1. Raw data (**a**) versus preprocessed data (**b**) of HPTLC profiles.

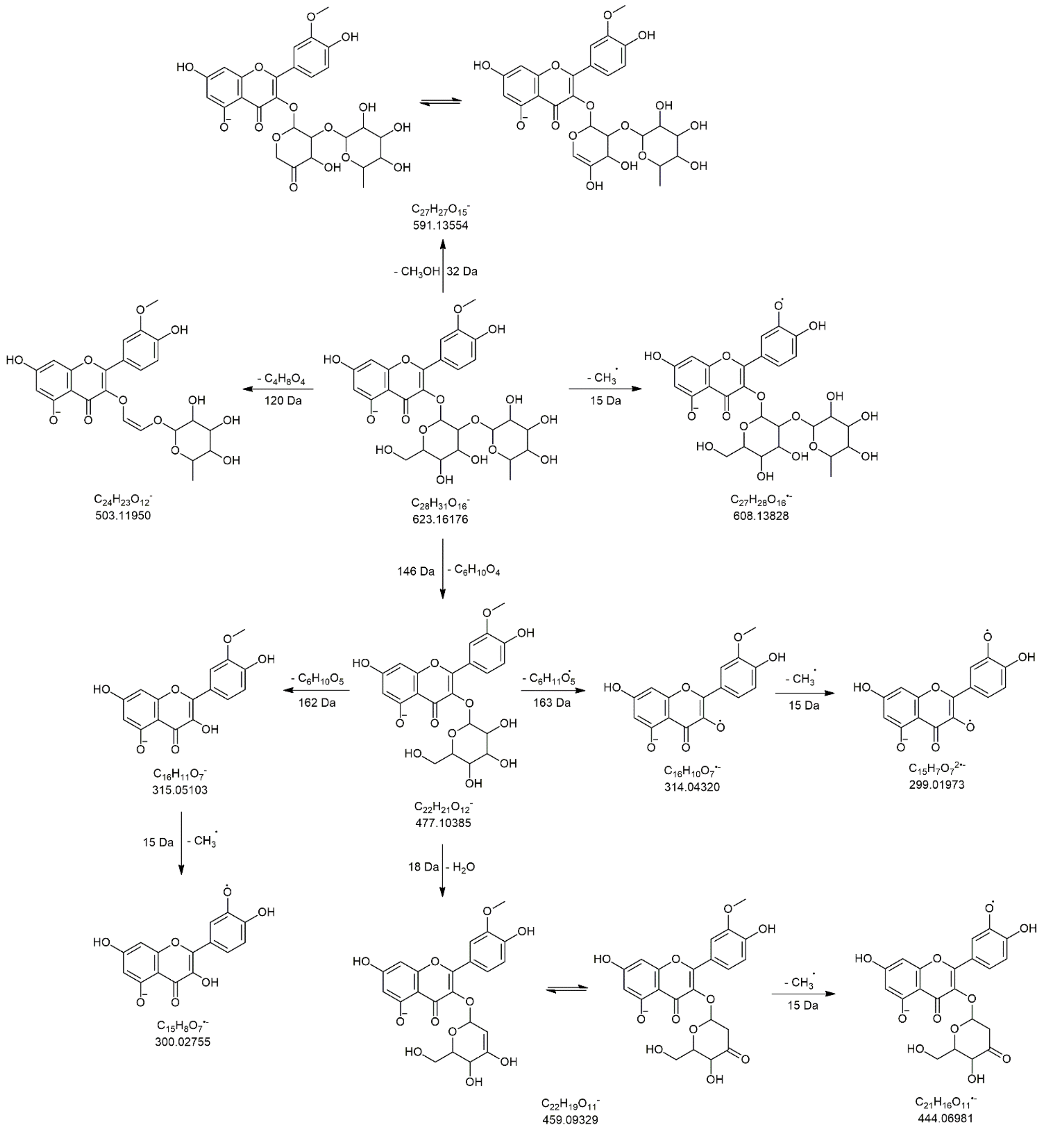

Figure S2. Proposed fragmentation pathway of compounds **14** and **19** (*m/z* 623).
